# Assessment of germline and somatic alterations in main candidate genes among patients with multiple primary melanoma

**DOI:** 10.1186/1479-5876-12-S1-O3

**Published:** 2014-05-06

**Authors:** Maria Colombino, Maria Cristina Sini, Amelia Lissia, Antonio Cossu, Vincenzo De Giorgi, Daniela Massi, Ignazio Stanganelli, Corrado Rubino, Antonella Manca, Grazia Palomba, Gerardo Botti, Corrado Caracò, Nicola Mozzillo, Paolo Antonio Ascierto, Giuseppe Palmieri

**Affiliations:** 1 Istituto di Chimica Biomolecolare, CNR, Sassari, Italy; 2 Istituto di Anatomia Patologica, AOU, Sassari, Italy; 3 Dipartimento di Dermatologia e Istituto di Anatomia Patologica, Università di Firenze, Firenze, Italy; 4 Istituto Tumori Romagna, Meldola, Italy; 5 Chirurgia Plastica, Universita di Salerno, Salerno, Italy; 6 Istituto Nazionale Tumori Fondazione Pascale, Naples, Italy

## Background

A series of patients with multiple primary melanoma (MPM) has been studied for assessing frequency and distribution of alterations in candidate genes involved in susceptibility (*CDKN2A*) and pathogenesis (*BRAF*, *cKIT*, *CyclinD1*) of such a disease.

## Methods

Two hundred and twenty-seven genomic DNA samples from paired synchronous and/or asynchronous tumour tissues of 106 MPM patients (92 cases with two, 13 with three, and 1 with four primary melanomas) were screened for somatic mutations in *BRAF* gene and FISH-based amplifications in *cKIT* and *CyclynD1* genes. For 87 (84%) MPM patients from our series, peripheral blood was available and germline DNAs was analyzed for mutations in *p16CDKN2A* and *p14CDKN2A* genes. All mutation analyses we performed by direct automated DNA sequencing. Family history for melanoma was defined according to standardized criteria.

## Results

At somatic level, *BRAF* mutations were identified in 108/227 (48%) primary melanoma tissues, whereas amplification of *cKIT* and *CyclinD1* genes was observed in 8/208 (4%) and 28/204 (14%) analyzed tissue samples, respectively. Considering all types of genetic events, paired samples presented a poorly consistent distribution of somatic alterations in same patients [55/106 (52%) discrepant cases). Among them, 35/106 (33%) patients presented discrepant MPM lesions according to the *BRAF* mutation status. Among the 87 MPM patients whose germline DNA was available, 8 (9%) of them showed different *CDKN2A* germline mutations: 7 in *p16CDKN2A* and 1 in *p14CDKN2A*. Assessment of family history for melanoma revealed that 13/87 (15%) patients presented at least one additional family member affected; a total of ≥ 3 melanomas in family was observed in 20/87 (23%) cases of our series. The *CDKN2A* germline mutations were found significantly more frequent in patients with familial history of melanoma (7/13; 54%) compared with patients without (1/74; 1.4%) (P<0.001); analogously, *CDKN2A* mutations were observed in 1/67 (1.5%) and 7/20 (35%) patients with 2 and 3 or more melanomas in family, respectively (P<0.001).

## Conclusions

The low consistency in mutation patterns at somatic level among MPM lesions from the same patients provide further evidence that melanomagenesis is heterogeneous and molecularly different cell types may participate to the development of multiple melanomas. Our findings on germline DNA indicate that occurrence of at least 3 melanomas (in patients or families) or familial recurrence of melanoma may represent strong indicators to address patients to *CDKN2A* mutation screening.

